# Deep learning segmentation of the choroid plexus from structural magnetic resonance imaging (MRI): validation and normative ranges across the adult lifespan

**DOI:** 10.1186/s12987-024-00525-9

**Published:** 2024-02-29

**Authors:** Jarrod J. Eisma, Colin D. McKnight, Kilian Hett, Jason Elenberger, Caleb J. Han, Alexander K. Song, Ciaran Considine, Daniel O. Claassen, Manus J. Donahue

**Affiliations:** 1 Department of Neurology, Behavioral and Cognitive Neurology, Vanderbilt University Medical Center, 1500 21 stAve South, Village at Vanderbilt, Suite 2600, Nashville, TN 37212 USA; 2 Department of Radiology and Radiological Sciences, Vanderbilt University Medical Center, Nashville, TN USA; 3 Department of Psychiatry and Behavioral Sciences, Vanderbilt University Medical Center, Nashville, TN USA; 4 Department of Electrical and Computer Engineering, Vanderbilt University, Nashville, TN USA

**Keywords:** Choroid plexus, Deep learning, Glymphatic, Segmentation, Cerebrospinal fluid, Neurofluids

## Abstract

**Background:**

The choroid plexus functions as the blood-cerebrospinal fluid (CSF) barrier, plays an important role in CSF production and circulation, and has gained increased attention in light of the recent elucidation of CSF circulation dysfunction in neurodegenerative conditions. However, methods for routinely quantifying choroid plexus volume are suboptimal and require technical improvements and validation. Here, we propose three deep learning models that can segment the choroid plexus from commonly-acquired anatomical MRI data and report performance metrics and changes across the adult lifespan.

**Methods:**

Fully convolutional neural networks were trained from 3D T_1_-weighted, 3D T_2_-weighted, and 2D T_2_-weighted FLAIR MRI using gold-standard manual segmentations in control and neurodegenerative participants across the lifespan (n = 50; age = 21–85 years). Dice coefficients, 95% Hausdorff distances, and area-under-curve (AUCs) were calculated for each model and compared to segmentations from FreeSurfer using two-tailed Wilcoxon tests (significance criteria: p < 0.05 after false discovery rate multiple comparisons correction). Metrics were regressed against lateral ventricular volume using generalized linear models to assess model performance for varying levels of atrophy. Finally, models were applied to an expanded cohort of adult controls (n = 98; age = 21–89 years) to provide an exemplar of choroid plexus volumetry values across the lifespan.

**Results:**

Deep learning results yielded Dice coefficient = 0.72, Hausdorff distance = 1.97 mm, AUC = 0.87 for T_1_-weighted MRI, Dice coefficient = 0.72, Hausdorff distance = 2.22 mm, AUC = 0.87 for T_2_-weighted MRI, and Dice coefficient = 0.74, Hausdorff distance = 1.69 mm, AUC = 0.87 for T_2_-weighted FLAIR MRI; values did not differ significantly between MRI sequences and were statistically improved compared to current commercially-available algorithms (p < 0.001). The intraclass coefficients were 0.95, 0.95, and 0.96 between T_1_-weighted and T_2_-weighted FLAIR, T_1_-weighted and T_2_-weighted, and T_2_-weighted and T_2_-weighted FLAIR models, respectively. Mean lateral ventricle choroid plexus volume across all participants was 3.20 ± 1.4 cm^3^; a significant, positive relationship (R^2^ = 0.54-0.60) was observed between participant age and choroid plexus volume for all MRI sequences (p < 0.001).

**Conclusions:**

Findings support comparable performance in choroid plexus delineation between standard, clinically available, non-contrasted anatomical MRI sequences. The software embedding the evaluated models is freely available online and should provide a useful tool for the growing number of studies that desire to quantitatively evaluate choroid plexus structure and function (https://github.com/hettk/chp_seg).

**Supplementary Information:**

The online version contains supplementary material available at 10.1186/s12987-024-00525-9.

## Background

The choroid plexus consists of a collection of fenestrated capillaries and epithelial cells that filter blood plasma down an osmotic gradient to secrete cerebrospinal fluid (CSF) in each of the brain’s four ventricles, with the majority of the choroid plexus volume residing in the atria of the lateral ventricles. The choroid plexus is widely believed to be the primary source of CSF production in the brain, producing CSF at a rate of 430–530 mL/day [1], and the choroid plexus has gained additional recent attention owing to its role as one of the most proximal components of the brain’s waste clearance system [2].

The choroid plexus structure and function has been well characterized from animal and post-mortem studies, but how choroid plexus structure and function change in the context of disease [3–5] and aging [6, 7] in humans is an area of active and emerging interest. In the context of aging, it has been shown that choroid plexus volume increases, and perfusion decreases, with advanced age [7, 8]. Diffusion-weighted magnetic resonance imaging (MRI) has also revealed that choroid plexus mean diffusivity increases, and fractional anisotropy decreases, with advanced age [8]. Increasing choroid plexus volume may relate to increasing severity of cognitive impairment in the spectrum of Alzheimer’s disease related disorders [3] and in support of this possibility, reduced choroid plexus metabolism from ^18^F-Fluorodeoxyglucose positron emission tomography (PET) has been reported in patients with Alzheimer’s disease compared to patients with amnestic mild cognitive impairment and healthy controls [9]. Perfusion-weighted arterial spin labelling MRI has been utilized further to characterize choroid plexus response to various pharmacological stimuli [10], which may aid in evaluating novel therapeutic delivery pathways or mechanisms.

However, one limitation to the advancement of neuroimaging studies of the choroid plexus is the lack of an accurate, automatic tool to segment the structure from anatomical images. Manual segmentations, as with other tissues, are impractical in large cohort studies, and the choroid plexus has varying appearances on standard MRI sequences due to its heterogeneous relaxometry characteristics [8], making manual segmentations an even more difficult process. Alisch et al. found that both the T_1_ and T_2_ relaxation times of the choroid plexus increase with advancing age [8], which affects the contrast of the choroid plexus on standard MRI sequences. For instance, this finding might suggest that the choroid plexus is more visible on T_1_-weighted and T_2_-weighted images for younger people, since the relaxometry of the choroid plexus contrasts with the surrounding CSF more in younger people.

Many neuroimaging software packages do not include segmentation options for the choroid plexus, and those that do include choroid plexus segmentation tools have been reported to have suboptimal performance in many applications [11]. Fully convolutional neural networks (FCNN) have shown state-of-the-art performance for segmentation of other brain structures [12], and recent work has proposed deep learning-based methods to segment the choroid plexus [11, 13, 14]. These methods rely on 3D magnetization-prepared-rapid-gradient-echo (MPRAGE) T_1_-weighted MRI to learn choroid plexus anatomical patterns, however, this approach may provide suboptimal contrast for choroid plexus visualization and quantification given limited contrast between hypointense CSF signal and normo-to-mildly hypointense choroid plexus signal. In addition to T_1_-weighted MRI, T_2_-weighted and T_2_-weighted FLuid Attenuated Inversion Recovery (FLAIR) MRI also are commonly acquired in both clinical and research neuroimaging environments and may yield differing segmentation accuracy, although this possibility has not been investigated rigorously.

In this study, we aim to develop and evaluate automated tools for segmenting the choroid plexus from three types of commonly acquired MRI sequences: T_1_-weighted, T_2_-weighted, and T_2_-weighted FLAIR; and to compare the results from these methods to gold-standard manual tracings and to commonly used neuroimaging analysis software, FreeSurfer [15, 16]. We also evaluate performance of these methods in an additional cohort of adult controls to report how the choroid plexus evolves across the adult lifespan, which will provide an exemplar for future clinical studies which may implicate the choroid plexus, such as Alzheimer’s disease, Parkinson’s disease, multiple sclerosis, and traumatic brain injury. The processing code is also made publicly available for free academic use.

## Methods

### Demographics

This study had two components. First, we developed and evaluated a deep learning algorithm using separate standard MRI sequences in a diverse cohort of adults (deliberately selected to span a range of ages and conditions) with the intent of providing a generalizable segmentation algorithm. Second, we applied the method to adult controls across the lifespan to provide an exemplar for how choroid plexus volume changes with age in a cross-sectional analysis.

Adult participants (n = 50 for model training; n = 98 for subsequent adult control lifespan analysis) provided informed, written consent in accordance with the Vanderbilt University Institutional Review Board (IRB) and the Declaration of Helsinki and its amendments. All participants were enrolled between February 2020 and July 2023. It is well-known that the brain atrophies with advancing age and in various neurological disorders, and with this atrophy comes ventricular enlargement and possibly choroid plexus hypertrophy [7, 8]. In order to make the proposed method as generalizable as possible, for algorithm training and development we deliberately enrolled a heterogeneous cohort of persons across the adult lifespan. Participants included controls and patients with mild cognitive impairment (MCI), Alzheimer’s disease, Parkinson’s disease, and Huntington’s disease. Inclusion criteria for control participants consisted of no history of cerebrovascular disease, anemia, psychosis, or neurological disorder including but not limited to prior overt stroke, sickle cell anemia, schizophrenia, bipolar disorder, Alzheimer’s disease, Parkinson’s disease, or multiple sclerosis. The presence of non-specific white matter lesions was not an exclusion criterion for controls, as these lesions become more prevalent with aging, and we sought our cohort to be generalizable and representative. Diagnosis of Alzheimer’s disease, mild cognitive impairment, Parkinson’s disease, or Huntington’s disease was made by a board-certified neurologist (DOC; experience = 15 years) using clinical criteria.

### Image acquisition

All participants underwent non-contrasted MRI at 3 Tesla with body coil radiofrequency transmission and 32-channel SENSE phased-array reception on a Philips Ingenia system (Philips Healthcare, Best, The Netherlands). Anatomical images consisted of: (i) 3D T_1_-weighted MPRAGE (TR = 8.1 ms; TE = 3.7 ms; field of view = 256 × 180 × 150 mm^3^; number of slices = 150; spatial resolution = 1.0 × 1.0 × 1.0 mm^3^; duration = 4 min 32 s), (ii) 2D T_2_-weighted FLAIR turbo-spin-echo (TR = 11,000 ms; TE = 120 ms; TI = 2800 ms; field of view = 230 × 184 × 144 mm^3^; number of slices = 29; spatial resolution = 0.57 × 0.57 × 4.0 mm^3^; duration = 1 min 39 s), and (iii) 3D T_2_-weighted turbo-spin-echo (TR = 2500 ms; TE = 331 ms; field of view = 250 × 250 × 189 mm^3^; number of slices = 242; spatial resolution = 0.78 × 0.78 × 0.78 mm^3^; duration = 4 min 8 s).

### Manual segmentation of the choroid plexus

Data utilized for manual segmentation of the choroid plexus consisted of 3D T_1_-weighted, 2D axial T_2_-weighted FLAIR, and 3D T_2_-weighted MRI from 50 participants. Ground truth choroid plexus segmentation was performed manually with final approval from a board-certified neuroradiologist (CDM; experience = 9 years). Additionally, for assessment of the inter-rater reliability of manual delineations of the choroid plexus, two additional raters manually segmented the choroid plexus following the same protocol as the primary rater in 10 participants from the machine learning training sample (see *Supplementary Materials*). In all cases, the manual delineation protocol was defined as follows: first, 2D axial T_2_-weighted FLAIR and 3D T_2_-weighted images were co-registered to 3D T_1_-weighted images using linear registration tools from the Advanced Normalization Tools (ANTs) software package [17]. Next, the contrast from all three co-registered images was utilized by the primary rater to generate a single choroid plexus segmentation for each participant, using the FMRIB Software Library (FSL) tool fsleyes for segmentation and to visualize all three images in the same space simultaneously [18]. This approach was chosen to make efficient use of the higher spatial resolution T_1_-weighted and T_2_-weighted scans as well as the intraventricular contrast afforded on the T_2_-weighted FLAIR. Given this process, a single ground truth segmentation was produced for each training subject. Manual segmentations focused on the choroid plexus in the atria of the lateral ventricles. We chose to focus on this region of the choroid plexus for several reasons. First, to limit biasing of the deep learning method, delineations were careful not to include partial voluming from nearby subcortical structures or periventricular white matter. The choroid plexus of the lateral ventricles minimizes the partial volumes effect, as the trigonum ventriculi contain the largest portion of the choroid plexus distinct from surrounding tissue [19]. It has been reported previously that across all four brain ventricles, more than half of the choroid plexus mass is located within the lateral ventricles [20].

### Automatic choroid plexus segmentation

Automatic choroid plexus segmentations were generated via a fully convolutional neural network model.

The machine learning model was designed following a U-NET architecture [21]. This architecture was chosen because of its proven success in medical image segmentation algorithms and consisted of an encoding and a decoding step. The encoding step was composed of three blocks each composed of two layers. The number of filters was set to 64 for the first block and doubled at each block thereafter. Each layer consised of a 3D convolution (kernel size = 3 × 3 × 3 voxels, stride = 1, and padding = 1), a batch normalization, followed by a rectified linear unit. Feature maps from each block were downsampled using a maximum pooling operation (kernel size = 2 × 2 × 2 voxels). The decoding step followed the same architecture, with each block dividing the number of filters by 2. Up-sampling between each decoding block was performed with a 3D transposed convolution (kernel size = 2 × 2 × 2 voxels, stride = 2 × 2 × 2 voxels, and no padding). The final segmentation map was obtained using a block composed of a 3D convolution operator (kernel size = 1 × 1 × 1 voxels, stride = 1 × 1 × x1 voxels, and no padding) followed by a hyperbolic tangent as the activation function. The model was trained on three separate datasets to compare performance across different MRI sequences (i.e., T_1_-weighted, T_2_-weighted, and T_2_-weighted FLAIR images). All images were registered non-linearly with ANTs software to the International Consortium for Brain Mapping-Montreal Neurological Institute (ICBM-MNI) 152 T_1_-weighted template [22]. Non-linear registration was utilized in this step in order to reduce morphological variability of the lateral ventricles and thus increase the inter-subject similarity of the choroid plexus appearance.

### Implementation details

**Fig. 1:**
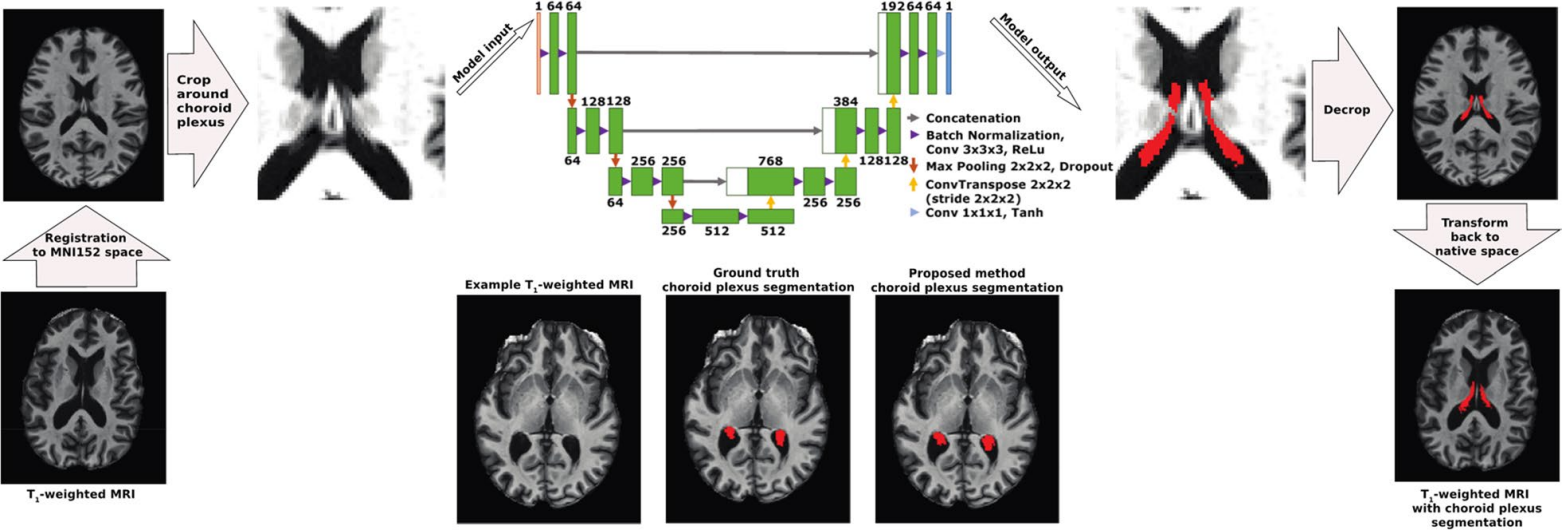
Overview of the processing pipeline of the anatomical magnetic resonance imaging (MRI) utilized in the proposed deep learning method. Examples are shown for a T_1_-weighted MRI, but this pipeline also was utilized for T_2_-weighted and T_2_-weighted FLuid-Attenuated Inversion Recovery (FLAIR) MRI. Input images were registered to MNI152 space and cropped around the choroid plexus based off a probabilistic atlas generated from ground truth manual choroid plexus segmentations. Cropped images were then used as training input for the 3D U-NET fully convolutional neural network. The number of inputs for each trained model was 1 and the number of output structures was 1 (i.e., choroid plexus). Cropped outputs were then decropped and inverse transformed to the native imaging space. Example images are shown from a 69 year old male with Parkinson’s disease. (MRI: magnetic resonance imaging; Conv: convolution; ReLu: rectified linear unit; Tanh: hyperbolic tangent)

A patch-based approach for training of the machine learning model was employed. Patches of 64 × 64 × 64 voxels were extracted from the MNI-registered images, and these patches were centered on random voxels from the choroid plexus probabilistic atlas that was generated from the average of the manual choroid plexus segmentations included in the training data set. In total, 41 overlapping patches from each participant were used to train the model. During the training phase, random flipping along the longitudinal fissure was implemented to increase the training sample size further. The network was trained using an ADAM [23] optimizer with a learning rate set to 10^–4^. A generalized Dice loss function was used to train the network [24]. Lastly, the segmentation mask patches were pieced back together in MNI space and transformed back to the native *T*_1_-weighted space using the inverse transformation and nearest-neighbor interpolation. An overview of the processing pipeline with a diagram of the 3D U-NET architecture is shown in Fig. 1.

For comparison to available algorithms, choroid plexus segmentation masks were generated using FreeSurfer’s standard segmentation procedure from T_1_-weighted MRI in all training subjects [15, 16]. Briefly, input images were skull-stripped and intensity corrected, and FreeSurfer’s *aseg* atlas was used to generate left and right choroid plexus labels. These labels were inverse transformed back to each subject’s native T_1_ space and combined to form one choroid plexus mask per subject. These masks were then compared to ground truth manual segmentations for statistical analysis. As a secondary and exploratory analysis, to assess test–retest reliability of the proposed machine learning methods, we analyzed consecutive T_1_-weighted MRI collected in 10 participants during different scan sessions within a 2 month time frame (see Aditional file 1).

Lastly, for the lifespan volumetry analysis, T_1_, T_2_, and T_2_-weighted FLAIR images were separately preprocessed as described previously, and the deep learning model for each corresponding MRI sequence was utilized to generate choroid plexus segmentations for all enrolled participants. From these segmentations in each participant’s native imaging space, the choroid plexus volume was calculated in cm^3^ .

### Statistical analyses

To evaluate the accuracy of the choroid plexus segmentation, we investigated how each model performed when trained with different sets of MRI sequences (i.e., T_1_-weighted, T_2_-weighted, and T_2_-weighted FLAIR images) compared to ground truth manual delineations. For each MRI sequence, a fivefold cross-validation scheme was implemented with 30 participants utilized in model training, 10 participants utilized in model validation, and 10 participants utilized in model evaluation. Pseudo-randomization was used to ensure the same participant groups across each modality-based model.

To verify the accuracy of the machine learning and FreeSurfer outputs, standard comparison metrics between the ground truth segmentation and machine learning output were calculated. The Dice-Sørensen coefficient, 95% Hausdorff distance, and area under curve (AUC) were calculated for each iteration of cross-validation and averaged across the iterations to produce representative metrics for each modality-based model. These metrics were then compared to FreeSurfer using two-tailed Wilcoxon tests.

As an exploratory analysis, we evaluated these performance metrics as a function of participant lateral ventricular volume to gain more understanding on how the machine learning models perform in different anatomical environments. Lateral ventricular volume was calculated from each participant’s T_1_-weighted MRI using the AssemblyNet software package [25]. Generalized linear models were utilized to separately regress performance metrics against model-testing participants’ lateral ventricular volume.

The intraclass correlation coefficients between the choroid plexus volume for each control participant from the three deep learning models and between each of the choroid plexus volumes for the training participants and their ground truth choroid plexus volumes, were calculated and descriptive statistics presented as Bland–Altman plots.

For the lifespan component of this study, a generalized linear model was utilized to regress the choroid plexus volume from each modality model against participant age. Sex was included as a covariate as well in this regression to account for previously found sex-dependence on choroid plexus volume [4], and total intracranial volume calculated from AssemblyNet was included as a covariate as well. The McFadden R^2^ values were calculated for each regression model.

The machine learning algorithm was implemented using the PyTorch Python library [26], and pre-processing, post-processing, and statistical analyses were implemented in Matlab [27]. All statistical analyses were implemented using the R software package [28]. All p-values were corrected with false discovery rate for multiple comparisons correction [29]. Significance criteria was defined as *p* < 0.05.

## Results

### Demographics: algorithm development

Participants (n = 50) included in the training, validation, and testing of the machine learning models ranged in age from 21 to 85 years, included 27 males and 23 females, and 29 control participants and 21 participants with neurodegeneration (Additional file 1: Table S1).

### Algorithm performance metrics

Performance metrics for each proposed machine learning model, and a previously available FreeSurfer algorithm, are reported in Table 1. The average Dice coefficients were 0.72, 0.72, and 0.74 for the T_1_-weighted, T_2_-weighted, and T_2_-weighted FLAIR models, respectively, while the average Dice coefficient for the FreeSurfer output applied to the T_1_-weighted image was 0.19. Two-tailed Wilcoxon tests revealed a significant difference in the Dice coefficient between the T_1_-weighted machine learning method and FreeSurfer, the T_2_-weighted machine learning method and FreeSurfer, and the T_2_-weighted FLAIR machine learning method and FreeSurfer (all p-values < 0.001).

**Table 1:** Performance metrics for each machine learning method and FreeSurfer using manual segmentations as the ground truth

Method	Sørensen–Dice Coefficient	95% Hausdorff Distance (mm)	AUC
Deep Learning from T_1_-weighted MRI	0.72 (*0.55–0.78*)***	1.97 (*1.00–6.71*)***	0.87 (*0.75–0.96*)***
Deep Learning from T_2_-weighted MRI	0.72 (*0.57–0.78*)***	2.22 (*1.00–16.6*)***	0.87 (*0.75–0.96*)***
Deep Learning from T_2_-weighted FLAIR MRI	0.74 (*0.61–0.80*)***	1.69 (*1.00–3.74*)***	0.87 (*0.74–0.96*)***
FreeSurfer from T_1_-weighted MRI	0.19 (*0.02–0.37*)	10.4 (*4.12–17.2*)	0.56 (*0.50–0.62*)

Values are shown as mean (range). Metrics for the machine learning-based methods were calculated from ten testing participants across five cross-validation iterations, whereas metrics for FreeSurfer were calculated from all 50 participants included in the algorithm development. *** indicates two-tailed Wilcoxon test revealed a significant difference between the machine learning method and FreeSurfer (p-value < 0.001)

The average 95% Hausdorff distances were 1.97, 2.22, and 1.69 mm for the T_1_-weighted, T_2_-weighted, and T_2_-weighted FLAIR models, respectively, and the average 95% Hausdorff distance for the FreeSurfer output was 10.4 mm. Two-tailed Wilcoxon tests revealed a significant difference in the 95% Hausdorff distance between the T_1_-weighted machine learning method and FreeSurfer, the T_2_-weighted machine learning method and FreeSurfer, and the T_2_-weighted FLAIR machine learning method and FreeSurfer (all p-values < 0.001).

The average AUCs were 0.87 for each of the models and the average AUC for the FreeSurfer output was 0.56. Two-tailed Wilcoxon tests revealed a significant difference in the AUC between the T_1_-weighted machine learning method and FreeSurfer, the T_2_-weighted machine learning method and FreeSurfer, and the T_2_-weighted FLAIR machine learning method and FreeSurfer (all p-values < 0.001).

**Fig. 2:**
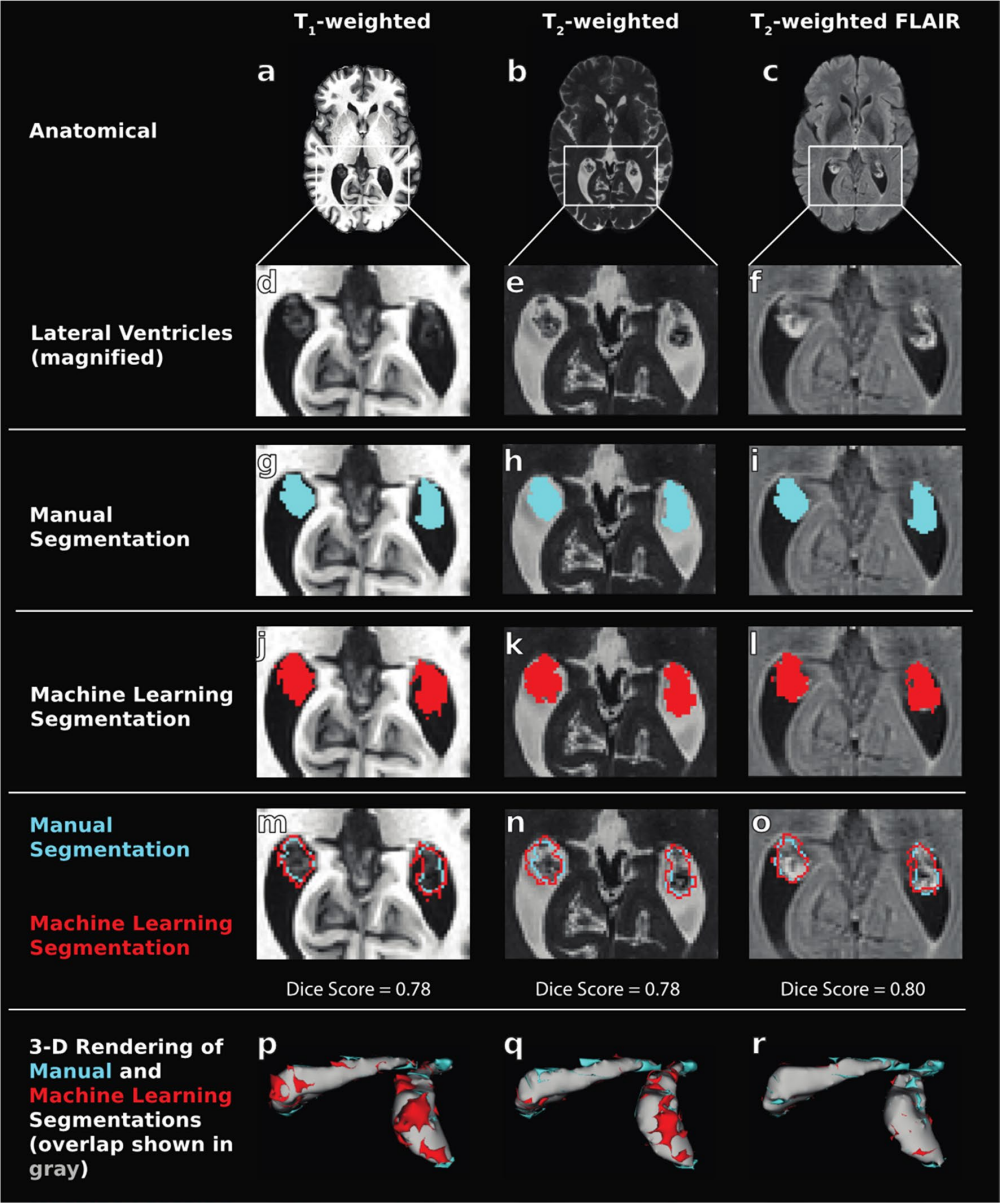
Example choroid plexus segmentations from machine learning models in a 53 year old male with mild cognitive impairment. From left to right, columns show results from T_1_-weighted images, T_2_-weighted images, and T_2_-weighted FLAIR images. The first row (panels **a**–**c**) shows the anatomical MRI sequence utilized in this study for deep learning training, and the second row (**d**–**f**) shows these same images magnified on the lateral ventricles where the majority of the choroid plexus resides. The remaining rows show the manual segmentations (**g**–**i**), machine learning output segmentations (**j**–**l**), and the overlay of these segmentations in axial slices (**m**–**o**) and 3D renderings (**p**–**r**) for each type of MRI contrast. The 3D renderings show the manual segmentation in blue (i.e., under-segmentation), the machine learning segmentation in red (i.e., over-segmetntation, and the overlap between the two in white. The Dice scores of each model (T_1_-weighted: 0.78, T_2_-weighted: 0.78, T_2_-weighted FLAIR: 0.80) are shown and reflect consistently accurate performance across MRI sequences. (MRI: magnetic resonance imaging; FLAIR: FLuid-Attenuated Inversion Recovery)

**Fig. 3:**
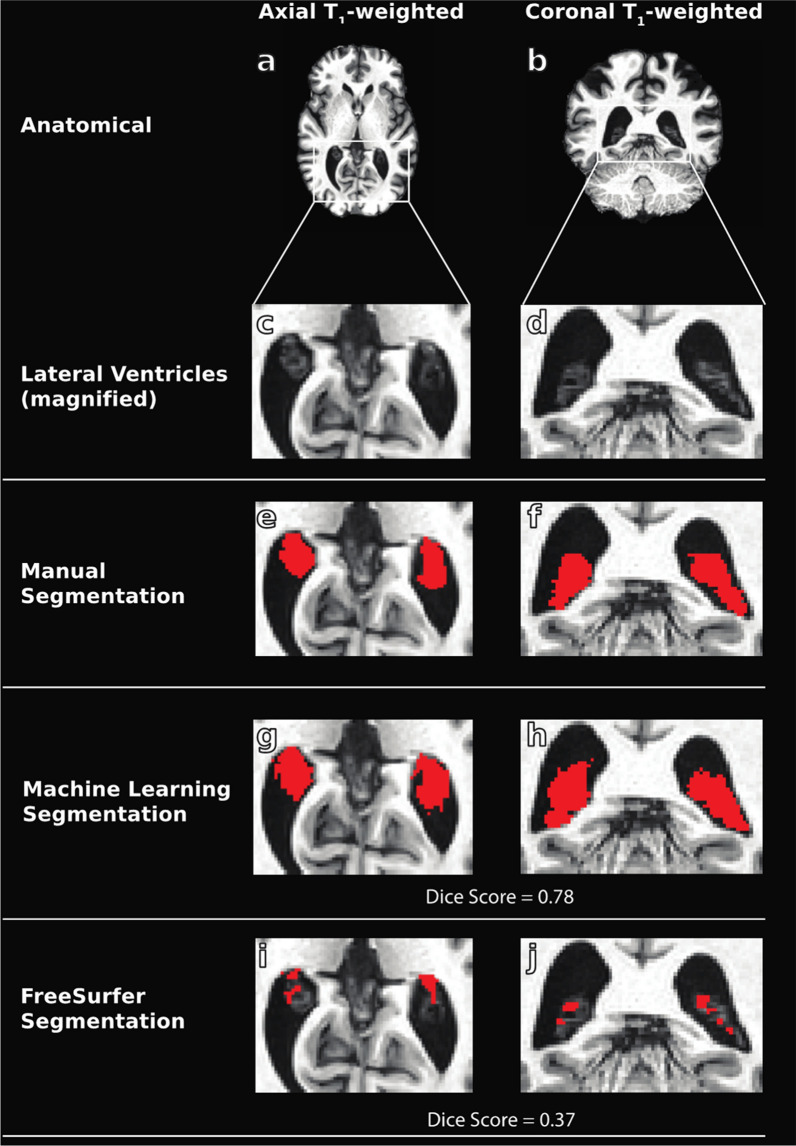
Example T_1_-weighted sequence from a 53 year old male with mild cognitive impairment (**a**–**d**) and manual tracings (**e**–**f**) utilized in training of the machine learning methods. Example outputs from the T_1_-weighted trained machine learning model (**g**–**h**) are shown compared to FreeSurfer segmentations (**i**–**j**). Dice scores are shown for machine learning and FreeSurfer outputs and reflect an improvement in segmentation accuracy for the proposed method

An example of each machine learning model output compared to ground truth and FreeSurfer choroid plexus segmentations from a 53-year-old male with MCI are shown in Figs. 2 and 3.

Finally, supplementary sub-analyses on inter-rater manual segmentation (ICC between all raters = 0.73) and inter-scan machine learning segmentation performance (ICC between consecutive segmentations = 0.99) are summarized in the *Supplementary Materials*.

**Fig. 4:**
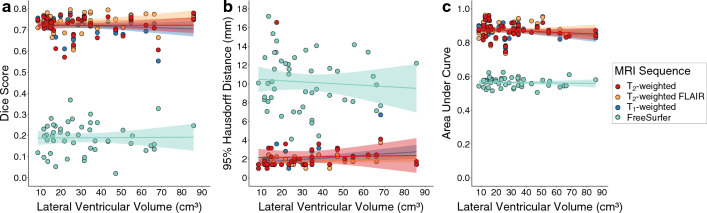
Regression plots for machine learning dice score (**a**), 95% Hausdorff distance (**b**), and AUC (**c**) against testing subject lateral ventricular volume. Overall, models performed consistently across lateral ventricular volume. The only model metric that was significantly related to lateral ventricular volume was the T_1_-weighted model’s 95% Hausdorff distance (ß value = 0.015; p-value = 0.05). (MRI: magnetic resonance imaging; FLAIR: FLuid-Attenuated Inversion Recovery)

We also investigated the relationship between model performance and lateral ventricular volume. Numerical results and graphical representations of these relationships are shown in Additional file 1: Table S2 and Fig. 4, respectively. For each MRI sequence, the lateral ventricular volume of the testing participant was not significantly related to the Dice coefficient (T_1_-weighted p-value: 0.44; T_2_-weighted p-value: 0.99; T_2_-weighted FLAIR p-value: 0.40). For the T_2_-weighted and T_2_-weighted FLAIR models, the lateral ventricular volume was not significantly related to the 95% Hausdorff Distance (T_2_-weighted p-value: 0.92; T_2_-weighted FLAIR p-value: 0.094); however, for the T_1_-weighted model, the lateral ventricular volume was positively related to the 95% Hausdorff Distance (p-value: 0.050). For each MRI sequence, the lateral ventricular volume was not significantly related to the AUC (T_1_-weighted p-value: 0.20; T_2_-weighted p-value: 0.35; T_2_-weighted FLAIR p-value: 0.69). For the FreeSurfer outputs, none of the metrics related to ventricular volume (Dice p-value: 0.99; 95% Hausdorff distance p-value: 0.44; AUC p-value: 0.69).

**Fig. 5:**
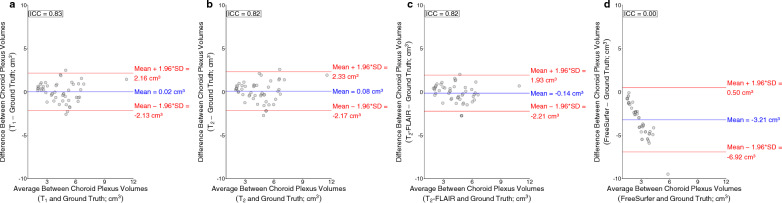
Bland-Altman plots for choroid plexus volumes generated from T_1_-weighted deep learning methods (**a**), T_2_-weighted deep learning methods (**b**), T_2_-weighted FLAIR deep learning methods (**c**), and FreeSurfer (**d**) compared to the ground truth manual segmentation volumes. The intraclass correlation coefficient between T_1_-weighted and ground truth choroid plexus volumes was 0.83, T_2_-weighted and ground truth choroid plexus volumes was 0.82, T_2_-weighted FLAIR and ground truth choroid plexus volumes was 0.82, and FreeSurfer and ground truth choroid plexus volumes was 0.00

Intraclass correlation coefficients were 0.83, 0.82, and 0.82 between T_1_-weighted deep learning choroid plexus volumes and ground truth choroid plexus volumes (Fig. 5a), T_2_-weighted deep learning choroid plexus volumes and ground truth choroid plexus volumes (Fig. 5b), and T_2_-weighted FLAIR deep learning choroid plexus volumes and ground truth choroid plexus volumes (Fig. 5c), respectively.

### Choroid plexus volume and age

Participants (n = 98) included in the assessment of choroid plexus volume across the adult lifespan ranged from 21 to 89 years of age and included 46 males and 52 females (Additional file 1: Table S3).

**Fig. 6:**
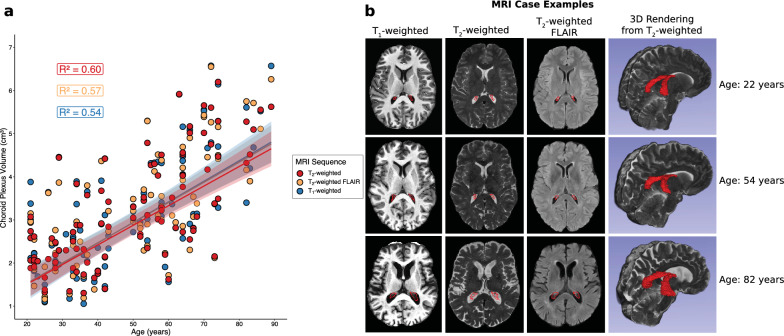
**a** Regression plot displaying choroid plexus volume against participants’ age for each MRI modality in adult controls. McFadden’s R^2^ values are reported for each regression model. **b** Case examples for younger, middle, and older-aged controls showing an increase in choroid plexus volume with age. Results show consistently across MRI types that choroid plexus volume increases with age across the adult lifespan. 3D renderings are shown from the T_2_-weighted segmentations to provide further support of this finding. (FLAIR: FLuid-Attenuated-Inversion-Recovery)

**Fig. 7:**
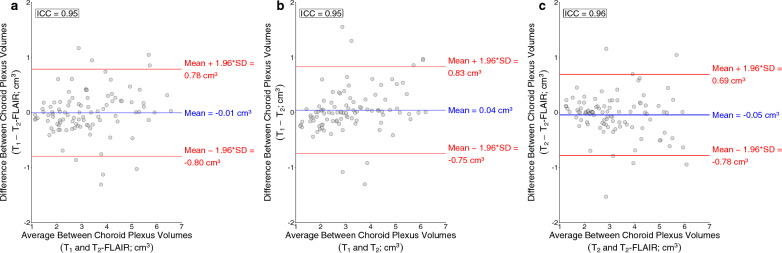
Bland-Altman plots for choroid plexus volumes generated from T_1_-weighted and T_2_-weighted FLAIR deep learning methods (**a**), T_1_-weighted and T_2_-weighted deep learning methods (**b**), and T_2_-weighted and T_2_-weighted FLAIR deep learning methods (**c**). The intraclass correlation coefficient between T_1_-weighted and T_2_-weighted FLAIR choroid plexus volumes was 0.95, T_1_-weighted and T_2_-weighted choroid plexus volumes was 0.95, and T_2_-weighted and T_2_-weighted FLAIR choroid plexus volumes was 0.96

Numerical and graphical results from these regression analyses are shown in Additional file 1: Table S4 and Fig. 6, respectively. For each MRI sequence, participant age was positively related to choroid plexus volume (all p-values < 0.001). Additionally, for each MRI sequence, participant sex was significantly related to choroid plexus volume, with males having a larger choroid plexus volume than females (T_1_-weighted p-value: 0.0012; T_2_-weighted and T_2_-weighted FLAIR p-values < 0.001). For each MRI sequence, total intracranial volume was not significantly related to choroid plexus volume (T_1_-weighted p-value: 0.094; T_2_-weighted p-value: 0.094; T_2_-weighted FLAIR p-value: 0.11). The McFadden’s R^2^ values for the T_1_-weighted, T_2_-weighted, and T_2_-weighted FLAIR regression models were 0.54, 0.60, and 0.57, respectively. Intraclass correlation coefficients between choroid plexus volumes were 0.95, 0.95, and 0.96 for T_1_-weighted and T_2_-weighted FLAIR deep learning methods (Fig. 7a), T_1_-weighted and T_2_-weighted deep learning methods (Fig. 7b), and T_2_-weighted and T_2_-weighted FLAIR deep learning methods (Fig. 7c). Representative choroid plexus volumes across the adult lifespan are included in Additional file 1: Table S3.

## Discussion

A deep learning method with 3D U-NET architecture was trained for automatic segmentation of the choroid plexus from standard anatomical MRI. Models were trained separately on three types of commonly-acquired images: T_1_-weighted, T_2_-weighted, and T_2_-weighted FLAIR MRI from a cohort of 50 participants across the adult lifespan and with differing levels of tissue atrophy. The findings of the study support improved automated segmentation of the choroid plexus using the proposed method compared to currently-available software, and also provides an exemplar of choroid plexus volumes, as a function of age, in controls that may provide a reference for studies in neurodegeneration. The software is also made freely available for academic use.

The three proposed deep learning methods were able to segment the choroid plexus with Dice coefficients, 95% Hausdorff distances, and AUC values comparable to those found in literature for choroid plexus segmentation [11, 13, 14, 30]. Zhao and colleagues report a mean dice score of 0.73 and a mean 95% Hausdorff distance of 1.87 utilizing a similar deep learning method with 3D U-NET architecture and 3D T_1_-weighted MRI from 10 healthy subjects for training data [11]. Yazdan-Panah and colleagues also propose a 3D U-NET method for automatic choroid plexus segmentation using 3D T_1_-weighted MRI from patients with multiple sclerosis (n = 97) and heatlhy controls (n = 44) for model training and report a mean dice score of 0.73 [14]. Lastly, Storelli and colleagues developed an automatic choroid plexus segmentation method utilizing a Gaussian Mixture Model from 3D T_2_-weighted FLAIR and 3D T_1_-weighted MRI in patients with multiple sclerosis (n = 55) and healthy controls (n = 60) and report mean dice scores of 0.63 in multiple sclerosis patients and 0.66 in healthy controls [30]. All training data in these studies were collected at 3.0 T. We expand on these methods by including additional anatomical MRI contrasts that are commonly acquired in clinical settings, specifically 3D T_2_-weighted and 2D T_2_-weighted FLAIR MRI, and a training dataset with diverse demographics with the goal of increasing the generalizability of the proposed methods. The proposed methods also showed improved performance compared to automatic segmentations from FreeSurfer across all calculated metrics, an important finding as many previous and ongoing studies utilize FreeSurfer for choroid plexus volumetric analyses [3, 8, 31]. FreeSurfer utilizes an atlas-based segmentation approach, whereby a manually labeled training set provided by the software is used to estimate probabilistic neuroanatomical labels for each voxel in the MRI volume registered to this atlas [15]. While this software has shown robust sensitivity for segmentation of many noncortical structures [15, 16], the results from this study and from Zhao et al. suggest that it may not be the most accurate for choroid plexus segmentation, possibly due to the inter-subject variation in choroid plexus structure [11]. Further, we found that most of the proposed models perform accurately independent of lateral ventricular volume. All model performance metrics had no significant relationship to testing subject lateral ventricular volume except the T_1_ model’s 95% Hausdorff distance. Observing the central plot in Fig. 4, it is possible that this relationship was driven by a statistical outlier. The observation that the other performance metrics were relatively stable in the presence of a variety of lateral ventricular volumes provides further support for the robustness of these models. In our test–retest analysis, the intraclass correlation coefficient between choroid plexus volumes in consecutively acquired T_1_-weighted MRI also was high (ICC = 0.99), again suggesting that the proposed method performs robustly in the context of repeated applications of the same algorithm to separate T_1_-weighted scans from the same subject (Additional file 1: Fig. S2).

Additionally, we applied these deep learning methods in a cohort of 98 adult controls across the adult lifespan and found a significant positive relationship between subject age and choroid plexus volume with all three methods. Intraclass correlation coefficients between these volumes were high, suggesting consistently accurate calculations of choroid plexus volumes between models in this cohort. Intraclass coefficients between training subjects’ ground truth choroid plexus volumes and deep learning choroid plexus volumes were also high, suggesting accurate segmentation performance from the proposed methods. We also reported normative ranges of choroid plexus volume across the adult lifespan and found an approximate 15% increase in choroid plexus volume with each decade of life on average across all MRI sequences, which agrees with previous reports from literature [7, 8]. We previously reported age-related increases in choroid plexus volume using similar methods as described in this study and age-related decreases in choroid plexus perfusion detected from arterial spin labeling MRI [7]. Sun and colleagues recently reported age-related increases in choroid plexus volume using manual delineations from T_1_-weighted MRI and enlarged stromal tissue in the choroid plexus of older subjects using ultrasmall superparamagnetic iron oxide (USPIO)-enhanced high resolution 2D gradient echo MRI at 7 Tesla [32]. Previous histopathological studies using hematoxylin–eosin staining have shown a thickened vascular wall and fibrotic stroma in the choroid plexus of elderly subjects as well [33], which could explain the enlarged volume on anatomical MRI.

While these methods showed robust results and provided findings that aligned with previous reports from literature, several factors should be considered when interpreting the results. The training data sample size included 50 participants. However, the chroid plexus was segmented using gold-standard manual segmentation by a radiologist and we chose a 3D U-NET architecture which has shown robust accuracy with limited data set samples [11, 21]. We also adopted a data augmentation strategy and utilized a patch-based approach and random flipping, which increased the training dataset from 50 to 4100 samples. Furthernore, we included participants in the training dataset with and without clinically diagnosed neurodegenerative diseases to increase generalizability. Additionally, the lifespan study reports on trends in choroid plexus volume with age, and participants are approximately equally distributed across the adult lifespan. However, this study was cross-sectional and not longitudinal (e.g., following the same participant over time) and also may be underpowered to infer small changes in choroid plexus volume over limited age epochs (e.g., a decade of life or less). Future work could expand on this cohort, using large data sets, to address these issues more rigorously. Lastly, when observing Table 1, Figs. 5, and 7 there is some variability in the calculated choroid plexus volumes compared to the ground truth volumes in all the proposed methods and when compared across the proposed methods. While this variability needs to be reduced in order to ensure accurate volume estimates, it is promising that the variability in Figs. 5a–c is considerably less than the variability produced from currently available automated segmentation approaches (Fig. 5d). It is also relevant to contextualize this variability with the variability that arises from manual segmentation procedures. In a separate analysis, we had two additional raters segment the choroid plexus in a subsample of the same 10 subjects using the same protocol described in the Methods. More details for the methods of this analysis can be found in the *Supplementary Materials*. The main finding from this analysis was that the variability produced from manual segmentations performed by separate raters was larger (overall ICC between 3 raters = 0.73) than the variability produced from the proposed automatic methods (ICC = 0.82). These analyses also stress the need to develop a more rigorous manual delineation protocol to assess the presence of ChP tissues in the lateral ventricles.

## Conclusion

We propose a deep learning segmentation method for automatic segmentation of the choroid plexus from the following standard anatomical MRI: T_1_-weighted, T_2_-weighted, and T_2_-weighted FLAIR. The proposed method performs similarly across these three commonly-acquired MRI sequences and improves segmentation accuracy compared to commercially available algorithms. Finally, we provide ranges for lateral ventricle choroid plexus volume across the adult lifespan, which should provide a useful exemplar for future work that aims to identify pathological aberrations in choroid plexus volume and function. The proposed method is also made freely available for academic use.

### Supplementary Information

**Additional file 1.** Additional figures and tables.

## Data Availability

The data that support the findings of this study are available from the corresponding author, MJD, upon reasonable request. Choroid plexus segmentation software is freely available for public use at: https://github.com/hettk/chp_seg

## Abbreviations

ANTs: Advanced Normalization Tools
AUC: Area under curve
CSF: Cerebrospinal fluid
FCNN: Fully convolutional neural network
FLAIR: Fluid attenuated inversion recovery
FSL: FMRIB Software Library
ICBM-MNI: International Consortium for Brain Mapping-Montreal Neurological Institute
IRB: Institutional Review Board
MCI: Mild cognitive impairment
MPRAGE: Magnetization-prepared-rapid-gradient-echo
MRI: Magnetic resonance imaging/images
PET: Positron emission tomography
TE: Echo time
TR: Repetition time
USPIO: Ultrasmall superparamagnetic iron oxide
