# Supplementary material for: Deep learning segmentation of the choroid plexus from structural magnetic resonance imaging (MRI): validation and normative ranges across the adult lifespan
Source: Fluids Barriers CNS. 2024 Feb 29;21:21. doi: 10.1186/s12987-024-00525-9 (PMC10903155; doi:10.1186/s12987-024-00525-9)
Supplement: Supplementary file 1 — Additional file 1. Additional figures and tables. [file 12987_2024_525_MOESM1_ESM.docx]

**ADDITIONAL FILE 1: MATERIALS**

**Additional file 1: Methods**

For assessment of the inter-rater reliability of manual delineations of the choroid plexus, two additional raters manually segmented the choroid plexus in the lateral ventricles following the same protocol as the primary rater described in the *Methods* section in 10 subjects from the machine learning training sample. In brief, these secondary raters generated segmentation masks from T_1_-weighted, and co-registered T_2_-weighted and T_2_-weighted-FLAIR, MRI resulting in one choroid plexus mask per subject in T_1_-weighted anatomical space. The intraclass correlation coefficient was calculated between each of the raters’ choroid plexus volumes (cm^3^).

For assessment of the test-retest reliability of the proposed machine learning methods, we retrospectively analyzed consecutive T_1_-weigthed MRIs collected in the same 10 subjects within a two month time frame. Four of the subjects had consecutive T_1_-weighted MRIs acquired with the following parameters: TR = 8.2 ms; TE = 3.8 ms; field of view = 256 x 240 x 180 mm^3^; number of slices = 180; spatial resolution = 1.0 x 1.0 x 1.0 mm^3^; duration = 2 minutes 45 seconds, compressed sensing: on. Six of the subjects had consecutive T_1_-weighted MRIs acquired with very similar albeit slightly different parameters: TR = 6.7 ms; TE = 3.0 ms; field of view = 240 x 240 x 170 mm^3^; number of slices = 170; spatial resolution = 1.0 x 1.0 x 1.0 mm^3^; duration = 3 minutes 2 seconds, compressed sensing: on. Images were analyzed using the same machine learning model trained from T_1_-weighted MRI, and the choroid plexus volume (cm^3^) was calculated from each outputted segmentation mask. The intraclass correlation coefficient was calculated between the first timepoint and second timepoint choroid plexus volumes.

**Additional file 1: Results**

The ICC between all three raters was 0.73, showing moderate reliability between manual segmentations of the choroid plexus. These results are shown in a Bland-Altman plot in Additional file 1: Figure 1.

The ICC between choroid plexus volumes in consecutively acquired T_1_-weighted MRI was 0.99. These results are shown in a Bland-Altman plot in Additional file 1: Figure 2.

**Additional file 1: Figures and Tables**

|  | Control | Neurodegeneration | Total |
| --- | --- | --- | --- |
| Sample size | 29 | 21 | 50 |
| Age range (years) | 21 – 82 | 38 – 85 | 21 – 85 |
| Age (years) | 47.6 ± 18.2 | 64.9 ± 11.9 | 54.9 ± 17.9 |
| Sex (% male) | 41.4 | 71.4 | 54.0 |

**Additional file 1: Table 1.** Participants used for model training. The cohort was deliberately selected to span the lifespan and to include healthy and neurodegenerative patients (Huntington’s disease, Parkinson’s disease, and Alzheimer’s disease) to create a generalizable algorithm. Each dataset included manual segmentations as supervised by a board-certified radiologist and a patch-based approach with random flipping was implemented to increase the training dataset from 50 to 4100 samples (see *Methods*). Ages are given as mean ± standard deviation.

| Segmentation Method | Dependent Variable | Intercept β₀ value | Intercept β₀ p-value | Lateral Ventricular Volume β₁ value | Lateral Ventricular Volume β₁ p-value | Coefficient of Determination (R²) | Model p-value |
| --- | --- | --- | --- | --- | --- | --- | --- |
| T₁-weighted | Sørensen–Dice Coefficient | **0.73** | **<0.001** | -2.9E-04 | 0.44 | 0.017 | 0.44 |
| T₂-weighted | Sørensen–Dice Coefficient | **0.72** | **<0.001** | 7.6E-06 | 0.99 | 1.1E-05 | 0.99 |
| T₂-FLAIR | Sørensen–Dice Coefficient | **0.73** | **<0.001** | 3.3E-04 | 0.40 | 0.022 | 0.40 |
| FreeSurfer | Sørensen–Dice Coefficient | **0.19** | **<0.001** | 3.9E-06 | 0.99 | 1.1E-06 | 0.99 |
| T₁-weighted | 95% Hausdorff Distance | **1.5** | **<0.001** | **0.015** | **0.050** | 0.095 | **0.043** |
| T₂-weighted | 95% Hausdorff Distance | **2.1** | **0.0013** | 2.8E-03 | 0.92 | 6.3E-04 | 0.92 |
| T₂-FLAIR | 95% Hausdorff Distance | **1.5** | **<0.001** | 6.6E-03 | 0.094 | 0.071 | 0.092 |
| FreeSurfer | 95% Hausdorff Distance | **11.0** | **<0.001** | -0.021 | 0.44 | 0.018 | 0.44 |
| T₁-weighted | AUC | **0.89** | **<0.001** | -5.0E-04 | 0.20 | 0.050 | 0.19 |
| T₂-weighted | AUC | **0.88** | **<0.001** | -3.6E-04 | 0.35 | 0.026 | 0.35 |
| T₂-FLAIR | AUC | **0.88** | **<0.001** | -1.9E-04 | 0.69 | 0.0052 | 0.69 |
| FreeSurfer | AUC | **0.57** | **<0.001** | -1.0E-04 | 0.69 | 0.0054 | 0.69 |

**Additional file 1: Table 2.** Results from regression models for performance metrics included in this study. Bold numbers represent beta coefficients with significant p-values (<0.05). For each stated dependent variable, the regression formula evaluated was: β₀ + β₁ x Lateral Ventricular Volume. (FLAIR: FLuid-Attenuated-Inversion-Recovery; AUC: area-under-curve; NA: not applicable).

|  | 20-39 years | 40-59 years | 60-89 years | Total |
| --- | --- | --- | --- | --- |
| Sample size | 38 | 28 | 32 | **98** |
| Age (years) | 28.7 ± 5.8 | 50.4 ± 6.7 | 70.9 ± 7.8 | **48.7 ± 19.1** |
| Sex (M/F) | 19/19 | 13/15 | 14/18 | **46/52** |
| T₁-weighted choroid plexus volume (cm³) | 2.29 ± 0.85 | 3.12 ± 0.89 | 4.36 ± 1.4 | **3.20 ± 1.4** |
| T₂-weighted choroid plexus volume (cm³) | 2.28 ± 0.67 | 3.15 ± 0.86 | 4.23 ± 1.2 | **3.16 ± 1.2** |
| T₂-weighted-FLAIR choroid plexus volume (cm³) | 2.31 ± 0.73 | 3.16 ± 0.93 | 4.33 ± 1.2 | **3.21 ± 1.3** |

**Additional file 1: Table 3.** Demographic and choroid plexus volumetric statistics for controls included in the application portion of this study. Values are reported as mean ± standard deviation where applicable.

| Segmentation Method | Intercept β₀ value | Intercept β₀ p-value | Sex β₁ value | Sex β₁ p-value | Age β₂ value | Age β₂ p-value | ICV β₃ value | ICV β₃ p-value | R² | Model p-value |
| --- | --- | --- | --- | --- | --- | --- | --- | --- | --- | --- |
| T₁-weighted | 0.60 | **0.039** | **0.66** | **0.0012** | **0.047** | **<0.001** | -0.0013 | 0.094 | 0.54 | **<0.001** |
| T₂-weighted | **0.61** | **0.027** | **0.75** | **<0.001** | **0.045** | **<0.001** | -0.0011 | 0.094 | 0.61 | **<0.001** |
| T₂-weighted FLAIR | **0.64** | **0.039** | **0.69** | **<0.001** | **0.046** | **<0.001** | -0.0012 | 0.11 | 0.57 | **<0.001** |

**Additional file 1: Table 4.** Results from regression models for choroid plexus volume across the adult lifespan included in this study for regression: Choroid Plexus Volume ~ β₀ + β₁ Sex + β₂ Age + β₃ ICV. Bold numbers represent beta coefficients with significant p-values (<0.05). (FLAIR: FLuid-Attenuated-Inversion-Recovery; ICV: total intracranial volume; NA: not applicable).


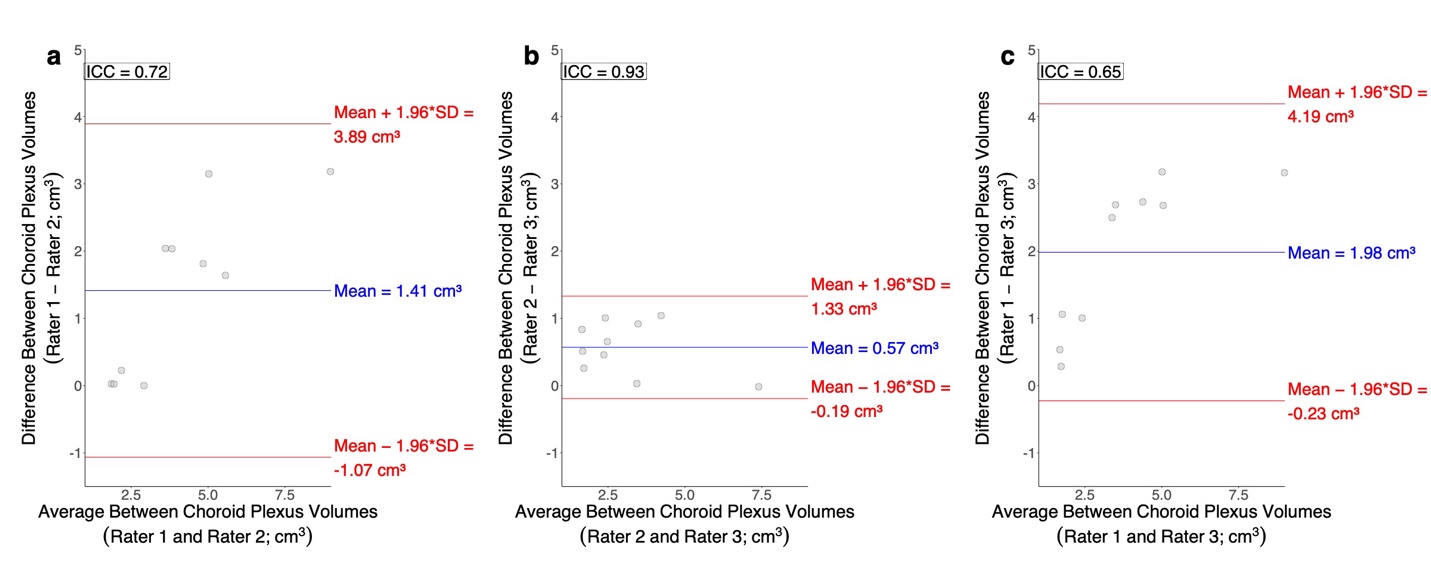


**Additional file 1: Figure 1.** Bland-Altman plots for choroid plexus volumes generated from raters 1 and 2 (a), raters 2 and 3 (b), and raters 1 and 3 (c). We observed an intraclass correlation coefficient between rater 1 and rater 2 choroid plexus volumes of 0.72, rater 2 and rater 3 choroid plexus volumes of 0.93, and rater 1 and rater 3 choroid plexus volumes of 0.65.


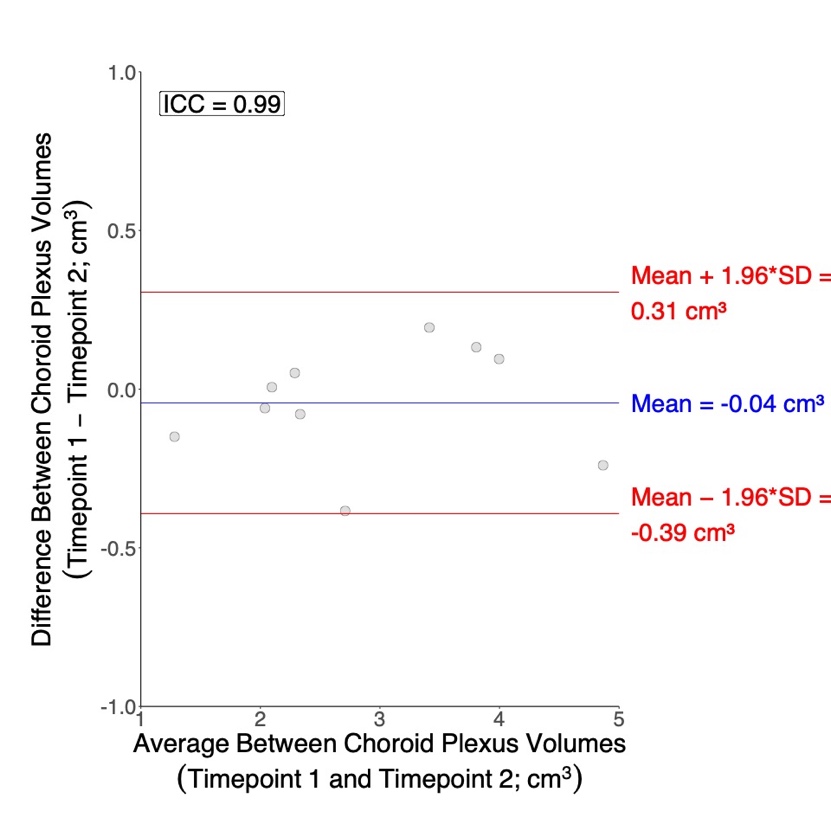


**Additional file 1: Figure 2.** Bland Altman plots for choroid plexus volumes generated from repeated T_1_-weighted MRI in the same 10 subjects. The intraclass correlation coefficient between choroid plexus volumes in consecutively acquired T_1_-weighted MRI was 0.99.
